# Pneumonic-type lung carcinoma with respiratory failure as the first manifestation: A case report and literature review

**DOI:** 10.1097/MD.0000000000045716

**Published:** 2025-11-14

**Authors:** Kaiyue Yue, Tianxing Zeng, Xiaoyan Qu, Famiao Zhang, Mingdong Zhao

**Affiliations:** a Department of Respiratory and Critical Care Medicine, The Second People’s Hospital of China Three Gorges University, Yichang, China.

**Keywords:** epidermal growth factor receptor, osimertinib, pneumonic-type lung carcinoma, respiratory failure, ultrasound-guided biopsy

## Abstract

**Rationale:**

Pneumonic-type lung carcinoma is a rare radiological lung cancer subtype. Its clinical and imaging manifestations are easily confused with pneumonia, leading to frequent misdiagnosis and mistreatment in clinical practice.

**Patient concerns:**

A 77-year-old female was admitted with “left-sided body pain for 1 week and head/facial pain for 2 days.” Initially lacking typical respiratory symptoms, she developed progressive respiratory failure requiring high-flow oxygen therapy after the initiation of anti-infective treatment.

**Diagnoses:**

Chest computed tomography revealed bilateral diffuse consolidation with air bronchograms. Ultrasound-guided percutaneous lung biopsy confirmed the diagnosis of invasive lung adenocarcinoma (non-mucinous). Genetic testing identified an epidermal growth factor receptor exon 19 deletion mutation (p.E746_A750del, variant allele frequency = 41.34%).

**Interventions:**

Supportive care, including anti-infectives, expectorants, and high-flow oxygen therapy, was administered upon admission. Following diagnosis, oral targeted therapy with osimertinib was initiated.

**Outcomes:**

The patient’s dyspnea gradually improved. Chest computed tomography performed 1 week later showed significant absorption of bilateral pulmonary shadows, and the oxygenation indices progressively normalized. Disease remission was sustained during the 2-month follow-up.

**Lessons:**

This critically ill patient presented with respiratory failure as the initial symptom, creating a high risk for misdiagnosis and mistreatment. Early recognition and definitive diagnosis are crucial for effective treatment. Bedside ultrasound-guided percutaneous biopsy is a viable diagnostic approach for critically ill patients.

## 1. Introduction

Lung cancer is one of the most common malignant tumors. According to 2022 global cancer statistics, there were nearly 2.5 million new lung cancer cases (12.4% of all cancers) and over 1.8 million deaths (18.7% of all cancer deaths), highlighting persistently high incidence and mortality rates.^[1]^ Pneumonic-type lung carcinoma (PTLC) is a special radiological subtype characterized by ground-glass opacities or pneumonia-like consolidation, often lacking distinct masses or nodules.^[2,3]^ Due to nonspecific clinical presentations and laboratory findings, PTLC is easily misdiagnosed as pneumonia or tuberculosis, leading to delays in diagnosis and treatment. This report presents a case of PTLC presenting initially with respiratory failure, along with a review of the relevant literature. The aim is to enhance early clinical recognition, reduce misdiagnosis and mistreatment, and improve patient survival and prognosis.

## 2. Case report

**Figure 1.**
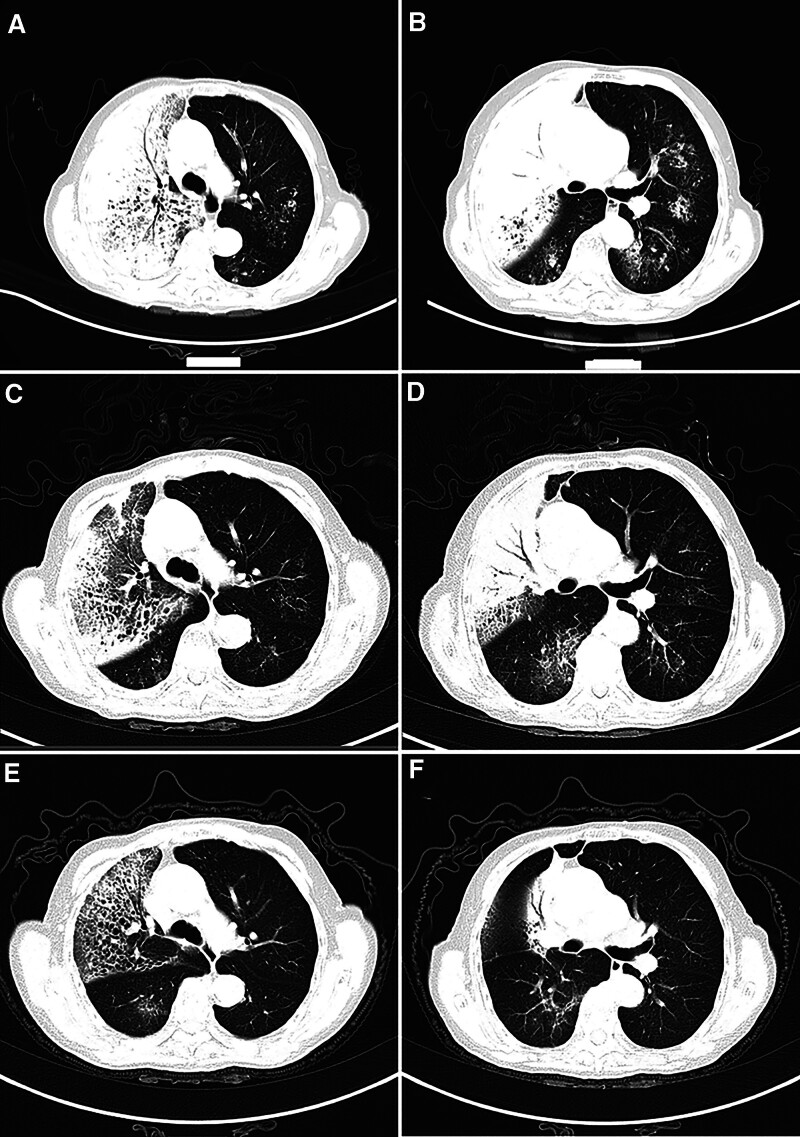
Imaging findings. (A and B) Initial chest CT upon admission showed multifocal patchy and large consolidations/increased opacities in both lungs, with right lung predominance. (C and D) Chest CT reexamined 1 week after osimertinib administration showed partial resolution/absorption of the extensive bilateral pulmonary opacities compared to the previous scan. (E and F) Follow-up chest CT was performed in our outpatient clinic after a course of regular/ongoing treatment. CT = computed tomography.

**Figure 2.**
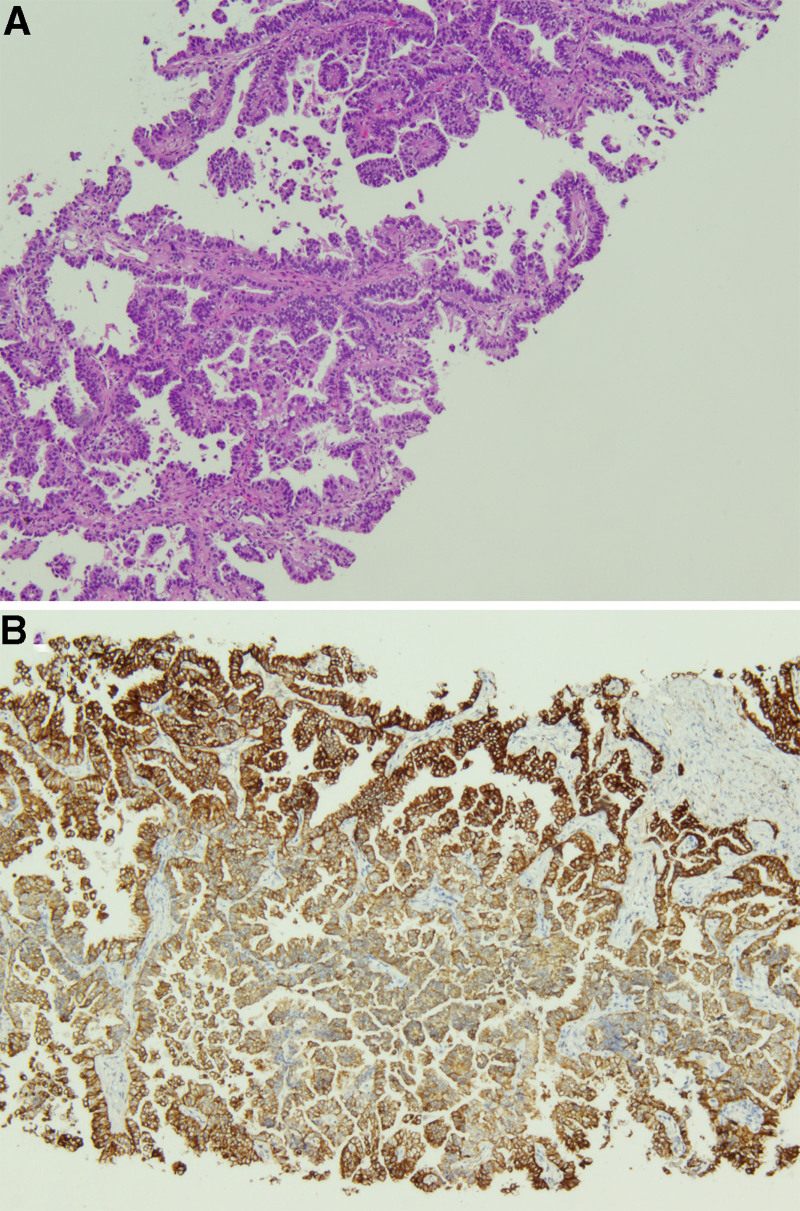
The percutaneous lung needle biopsy pathology revealed invasive adenocarcinoma (non-mucinous type) with a predominantly papillary growth pattern (A and B).

A 77-year-old female was admitted to the Neurology Department on November 2, 2024, for “sudden left-sided body pain for 1 week and head/facial pain for 2 days.” One week prior, she developed sharp, paroxysmal left-sided body pain after catching a cold, which did not affect her sleep and was not accompanied by nausea, vomiting, palpitations, or chest tightness. Two days before admission, she experienced persistent pain in the right maxillary region, significant fatigue, poor appetite, and dyspnea without tinnitus, hearing loss, or slurred speech. Initial diagnosis was “herpes zoster neuralgia.” Admission chest computed tomography (CT) suggested severe bilateral pulmonary infection, and follow-up after treatment was recommended (Fig. 1A and B). She was subsequently transferred to our department for further management. The patient’s medical history was unremarkable, with no history of smoking or family history of hereditary diseases or tumors. Her personal, menstrual, and marital histories were nonspecific. Physical examination revealed a temperature of 36.7℃, pulse of 96 beats/min, respiration of 21 beats/min, and blood pressure of 122/73 mm Hg (1 mm Hg = 0.133 kPa). There was slight cyanosis of the lips and mouth, red papules on the left side of the chest, obvious tenderness, thick breath sounds in both lungs, and wet rales in the right upper lung. Heart sounds were normal, and no murmurs were detected in the auscultation area of the heart valves. The abdomen was flat, soft throughout, and nontender, with no rebound pain. The bladder was not distended, and neither kidney was palpable. There was no percussion pain in the renal region and no edema in either lower limb. After admission, blood gas analysis: PH 7.37, arterial partial pressure of oxygen 54 mm Hg, partial pressure of carbon dioxide 46 mm Hg, oxygen saturation 87%, oxygenation index 89, blood routine + ultrasensitive C-reactive protein: leukocyte 6.5 × 10^9^/L, neutrophil percentage 81.6%, lymphocyte percentage 9.3%, monocyte count 0.67 × 10^9^/L, normal C-reactive protein. Interleukin-6, erythrocyte sedimentation rate, and calcitoninogen levels were the results of the lung cancer special test 5: gastrin releasing peptide precursor, 69.97 pg/mL; cytokeratin 19 fragment, 3.83 ng/mL; carcinoembryonic antigen, neuron-specific enolase, and squamous epithelial cell carcinoma antigen were normal. Sputum exfoliative cell examination revealed suspicious malignant cells. Combined with the patient’s medical history and examination results, a preliminary diagnosis of community-acquired pneumonia was made, and empirical anti-infective treatment with piperacillin sodium and tazobactam sodium was administered, along with doxophylline to calm asthma, methylprednisolone hormone for anti-inflammatory treatment, and sustained high-flow oxygen therapy (with the highest concentration of oxygen up to 100%) to maintain the oxygen saturation level. Blood gas analysis showed a pH of 7.40, arterial partial pressure of oxygen of 54 mm Hg, partial pressure of carbon dioxide of 49 mm Hg, oxygen saturation of 88%, and an oxygenation index of 60. Blood gas analysis did not show any improvement, and the patient’s dyspnea and decreased activity endurance symptoms did not significantly reduce. On November 6, 2024, an ultrasound-guided percutaneous biopsy of the right lung tissue was performed. Pathology: invasive adenocarcinoma (non-mucinous type) with a predominantly papillary growth pattern (Fig. 2A and B). Lung tissue genetic testing was performed for the epidermal growth factor receptor (EGFR) exon 19 deletion mutation (EGFR NM_005228_5 exon19 c.2236_2250del p.E746_A750del, variant abundance 41.34%). As the patient was unable to tolerate PET-CT, bone ECT, and cranial nuclear magnetism for systemic evaluation, bedside ultrasound was performed, and no distant organ or lymph node metastasis was observed. Final diagnosis: right lung malignant tumor, cT4N0M1a, stage IVA. Treatment was administered with osimertinib (80 mg 1 time/d orally) anti-tumor targeting therapy. On November 25, 2024, chest CT was reviewed to see a large number of shadows in both lungs partially absorbed compared with the previous one (Fig. 1C and D), and the blood gas analysis and symptoms had gradually improved. On November 29, 2024, blood gas analysis was performed to assess the recovery of oxygenation (Table 1), with an oxygen saturation level of 98% and an oxygenation index of 330. The patient’s condition was stabilized, and he was discharged from the hospital. A chest CT performed on January 1, 2025, suggested that the right lung lamellar shadow and bilateral lung infection had improved (Fig. 1E and F). After the patient was treated with targeted therapy, the symptoms of respiratory failure gradually improved, and follow-up chest CT showed that the large shadows in both lungs were significantly reduced compared with before. The patient’s condition is stable and is still under treatment.

**Table 1:** Changes in arterial blood gas (ABG) analysis during hospitalization.

Date	pH	PaO_2_ (mm Hg)	PaCO_2_ (mm Hg)	FiO_2_ (%)	P/F	SaO_2_ (%)
November 3 (O₂ 10 L/min)	7.37	54	46	61	89	87
November 6 (HFNC 55 L/min)	7.40	54	49	90	60	88
November 8 (HFNC 55 L/min)	7.40	49	52	95	52	84
November 26 (O₂ 3 L/min)	7.35	68	58	33	206	92
November 29 (O₂ 3 L/min)	7.31	109	58	33	330	98

## 3. Discussion

PTLC is a distinct form of lung cancer, predominantly adenocarcinoma in pathology.^[4]^ Its pathological hallmark is tumor cells growing along alveolar walls while preserving alveolar architecture, resulting in imaging features mimicking pneumonia and frequent misdiagnosis.^[5]^ Typical clinical symptoms include cough with copious white mucoid sputum, with or without dyspnea, often lacking specificity.^[6,7]^ This case was unusual because the patient initially presented with respiratory failure without prominent cough or sputum, posing a significant diagnostic challenge.

Initially misdiagnosed as community-acquired pneumonia based on symptoms, signs, and CT findings, critical reevaluation was prompted by the absence of typical infectious symptoms (chills, fever, purulent sputum), lack of response to empirical antibiotics, and elevated tumor markers despite normal inflammatory markers. The patient’s critical condition precluded standard invasive biopsies (bronchoscopy and CT-guided). The strategic use of bedside ultrasound-guided percutaneous biopsy, targeting the consolidation adjacent to the pleura, was instrumental in achieving a definitive diagnosis of lung adenocarcinoma.

Emerging evidence indicates that tumor microenvironment heterogeneity is a key driver of lung cancer initiation, spread, treatment response, and relapse.^[8]^ Of particular relevance to pneumonic-type presentations is the spatial heterogeneity of the intratumoral microbiota.^[9,10]^ Recent studies show that microbial communities can modulate local inflammation, mucin dynamics, epithelial plasticity, and immune tone, thereby shaping radiologic consolidation patterns and correlating with progression risk and outcomes.^[11]^ The rising focus on cancer microbiomes suggests these factors may influence both the atypical clinical course and prognosis of PTLC, and they warrant routine consideration in differential diagnosis and longitudinal monitoring.^[12]^

Multi-omics (genomics, transcriptomics, epigenomics, proteomics, metabolomics, and microbiomics) provides a systems-level view of PTLC biology, enabling: deconvolution of cellular ecosystems (cancer/immune/stromal); nomination of druggable pathways beyond EGFR; and remodeling of response monitoring using circulating tumor DNA and proteo-metabolomic signals.^[13,14]^ Integrated multi-omics has accelerated target discovery and precision pharmacology in cancer and is directly applicable to PTLC, where atypical radiologic phenotypes may reflect distinct molecular circuits.^[15]^

Clinically and radiologically, PTLC closely resembles pneumonia.^[16]^ However, certain CT features can aid in differentiation.^[17]^ Zhang et al^[18]^ identified irregular air bronchograms, vacuole signs, and satellite lesions as key discriminators. Huo et al^[7]^ reported that PTLC more frequently exhibits bulging interlobar fissures, low-attenuation areas within the consolidation, vacuole signs, and CT angiogram signs. The initial CT (Fig. 1A), extensive consolidation with classic air bronchograms and tree-in-bud signs was evident. These findings align with the tumor growth pattern along the alveolar and bronchiolar walls, preserving the structure but causing consolidation. Bronchial invasion manifests as rigidity, distortion, narrowing, truncation, cyst formation, and honeycombing,^[16]^ consistent with Akira et al description of PTLC.^[19]^ Therefore, chest CT provides vital clues for the diagnosis of PTLC. When characteristic imaging features persist despite adequate anti-infective therapy, PTLC should be suspected. For such patients, bronchoscopic biopsy or percutaneous lung biopsy should be pursued aggressively for definitive diagnosis.^[20]^ In critically ill patients unable to tolerate invasive procedures, repeated sputum cytology is essential to detect malignant cells, aiming for early diagnosis and treatment to prevent delays. This case exemplifies a successful diagnosis via bedside ultrasound-guided biopsy in a critically ill, procedure-intolerant patient.

Osimertinib is a 3rd-generation, irreversible EGFR-tyrosine kinase inhibitor that selectively inhibits sensitizing mutations (including exon 19 deletion) and T790M while sparing wild-type EGFR, and it penetrates the blood–brain barrier.^[21]^ In PTLC with EGFR 19del, rapid reductions in inflammatory-appearing consolidations likely reflect direct suppression of mutant-EGFR signaling (RAS–RAF–MEK–ERK; PI3K–AKT–mTOR) and secondary tumor microenvironment normalization, consistent with swift improvements in oxygenation and imaging.^[22]^ Parallel mechanistic lines underscore the therapeutic relevance of oxidative stress and matrix/PI3K–AKT control in lung cancer: sanguinarine induces ROS-mediated apoptosis and growth inhibition,^[23]^ while tetrandrine can inhibit malignant progression via down-regulating MMP1 and restraining PI3K/AKT signaling.^[24]^ Although these natural compounds are not standard of care, they highlight redox and extracellular-matrix/AKT axes as complementary vulnerabilities worth mechanistic discussion in PTLC.

Growing data link specific genomic lesions to TMB levels, immune contexture, and prognosis in lung cancer.^[25]^ Incorporating TMB and genomic-instability markers into PTLC evaluation may refine prognostic stratification and identify patients who could benefit from immunotherapy or combinatorial strategies, particularly when EGFR-targeted therapy fails.^[26]^

PTLC is uncommon and often diagnosed late; therefore, early recognition and treatment are critical. Clinicians should promptly reevaluate for PTLC when “pneumonia-like” consolidations or extensive ground-glass opacities persist despite adequate antibiotics, typical infectious features are absent (fever, purulent sputum), and inflammatory markers are unremarkable. Suspicious CT clues include persistent lobar/segmental consolidation with irregular air bronchograms, small low-attenuation foci/vacuoles, bulging fissures, a CT-angiogram sign, satellite lesions, or a non-resolving tree-in-bud pattern. When PTLC is strongly suspected, pursue pathological confirmation while prioritizing safety and avoiding unnecessary surgery; in unstable patients who cannot undergo bronchoscopic or CT-guided biopsy, bedside ultrasound-guided pleura-adjacent (percutaneous) biopsy provides a practical alternative. Early molecular profiling (EGFR and other drivers) is encouraged, as a brisk response to targeted therapy can be both therapeutic and diagnostically informative. Ultimately, an integrated differential (synthesizing clinical course, laboratories, imaging, and histology) helps distinguish infection and organizing pneumonia from invasive mucinous adenocarcinoma or lymphoma, thereby shortening time to definitive management and improving outcomes.

## Acknowledgments

We thank the efforts and contributions of the reported patients and all the clinical staff in this study.

## Author contributions

**Conceptualization:** Mingdong Zhao.

**Formal analysis:** Xiaoyan Qu.

**Supervision:** Famiao Zhang.

**Writing – original draft:** Kaiyue Yue.

**Writing – review & editing:** Tianxing Zeng.
